# Chiral Systems Made from DNA

**DOI:** 10.1002/advs.202003113

**Published:** 2021-01-21

**Authors:** David Winogradoff, Pin‐Yi Li, Himanshu Joshi, Lauren Quednau, Christopher Maffeo, Aleksei Aksimentiev

**Affiliations:** ^1^ Center for the Physics of Living Cells University of Illinois at Urbana–Champaign Urbana IL USA; ^2^ Department of Physics University of Illinois at Urbana–Champaign Urbana IL USA; ^3^ Beckman Institute for Advanced Science and Technology University of Illinois at Urbana–Champaign Urbana IL USA

**Keywords:** DNA origami, liquid crystals, nanotechnology, plasmonics, self‐assembly

## Abstract

The very chemical structure of DNA that enables biological heredity and evolution has non‐trivial implications for the self‐organization of DNA molecules into larger assemblies and provides limitless opportunities for building functional nanostructures. This progress report discusses the natural organization of DNA into chiral structures and recent advances in creating synthetic chiral systems using DNA as a building material. How nucleic acid chirality naturally comes into play in a diverse array of situations is considered first, at length scales ranging from an individual nucleotide to entire chromosomes. Thereafter, chiral liquid crystal phases formed by dense DNA mixtures are discussed, including the ongoing efforts to understand their origins. The report then summarizes recent efforts directed toward building chiral structures, and other structures of complex topology, using the principle of DNA self‐assembly. Discussed last are existing and proposed functional man‐made nanostructures designed to either probe or harness DNA's chirality, from plasmonics and spintronics to biosensing.

## Graphical abstract

This progress report discusses the natural organization of DNA into chiral structures and recent advances in creating synthetic chiral systems using DNA as a building material. The topics covered include chirality of biological DNA systems at all scales, liquid crystal phases of dense DNA solutions, programmable chirality in self‐assembled DNA nanostructures, and DNA‐based plasmonic and spintronic systems.

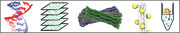

## 1 Introduction

Within all living organisms, virtually all amino acids are left‐handed and all sugars are right‐handed, a striking feature referred to as homochirality. Among the three types of biomolecules produced by the polymerization of homochiral components—proteins, RNA, and DNA—the latter stands out as its secondary structure is predominately a right‐handed double‐helix, whereas proteins and RNA can fold upon themselves in a number of different ways. Furthermore, the DNA of bacteria, archaea, and eukaryotes is systematically underwound^[^ ^1^ ^]^ and therefore is chiral in a more global sense as well.

On the opposite spectrum of scales, chiral structures are omnipresent in engineering, from bolts and screws to stairwells and windmills to even our own two hands that are of opposite chirality. More likely than not, you are reading this text because liquid crystals in your computer display adopt a different degree of twist along the path of light in a pattern prescribed by the local electric field.

What happens when the intrinsically chiral molecule, the DNA itself, is used to build devices that operate at the nanoscale? How does the chirality of the building blocks propagate across the scales and what bearing does such a choice of material have on the function of the resulting assemblies? Finally, does the chiral nature of DNA offer any benefits for design of devices that utilize chirality for their operation?

We begin answering these questions by examining first the role the chirality of DNA plays in its native environment—the interior of a biological cell. Moving on, we review recent efforts to understand how the chirality of DNA molecules prescribes the properties of the liquid crystalline phases that DNA forms at high concentration. Following that, we examine chiral effects in the context of man‐made nanostructures that use DNA as a building material. Lastly, we discuss how the chirality of DNA‐containing structures can be applied to realize specific functions, including bending light, sensing, splitting water, storing memory, and drug delivery. We conclude with a summary and a list of open questions.

## 2 Chirality in Biological DNA Systems

**Figure 1:**
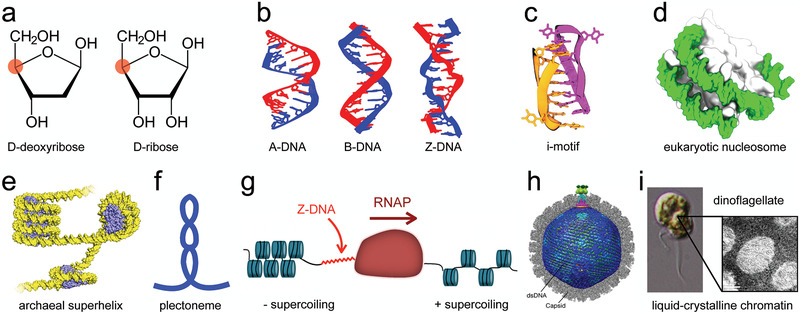
Chirality in biological nucleic acid systems. a) Stereochemical representations of d‐deoxyribose and d‐ribose. Red dots indicate the chirality centers. b) Dodecamers of double‐stranded A‐DNA, B‐DNA and Z‐DNA, based on PDB IDs 5MVT,^[^ ^29^ ^]^ 1RVI,^[^ ^30^ ^]^ and 1JES,^[^ ^31^ ^]^ respectively. c) An intercalated DNA structure, referred to as an “i‐motif,” based on PDB ID 2MRZ.^[^ ^32^ ^]^ d) Eukaryotic nucleosome based on PDB ID 1AOI.^[^ ^33^ ^]^ e) Archaeal superhelix based on PDB ID 5T5K.^[^ ^34^ ^]^ Reproduced with permission.^[^ ^34^ ^]^ Copyright 2017, The Authors, published by American Association for the Advancement of Science. f) Schematic of a plectoneme. g) DNA supercoiling generated during transcription by RNA polymerase (RNAP). Adapted with permission.^[^ ^35^ ^]^ Copyright 2012, Elsevier. h) Cryo‐EM structure of the herpes simplex virus 1 capsid (gray) and left‐handed‐spooled dsDNA genome (blue). Reproduced with permission.^[^ ^36^ ^]^ Copyright 2019, Springer Nature. i) A unicellular dinoflagellate and TEM photomicrograph of its chromosomes. Adapted with permission.^[^ ^37^ ^]^ Copyright 2010, American Society for Microbiology; Adapted with permission.^[^ ^38^ ^]^ Copyright 2013, Cell Press.

The chirality of nucleic acids derives from the compositional asymmetry of the sugars forming the DNA and RNA backbones: d‐deoxyribose and d‐ribose, **Figure** **1**a. In nature, DNA is predominately found to adopt the structure of a double‐stranded helix of right‐handed B‐form, Figure 1b. Under extreme conditions and for specific nucleotide sequences, DNA can also adopt a right‐handed A‐form or a left‐handed Z‐form, where “Z” stands for the zigzag path of the strands' backbone, Figure 1b. A‐form can be found in ethanol solutions^[^ ^2^, ^3^ ^]^ and at low humidity,^[^ ^4^ ^]^ and can be more favorable for sequences of a high CG content.^[^ ^5^ ^]^ Z‐form becomes more likely under high salt concentrations,^[^ ^6^ ^]^ and for d(CG)_*n*_ sequences found near the sites of transcription initiation.^[^ ^7^ ^]^

Single‐stranded DNA (ssDNA) exists inside cells, but it is mostly sequestered by binding proteins that largely prevent the formation of ssDNA secondary‐structure to help ensure the genome's integrity.^[^ ^8^ ^]^ Unlike DNA, RNA can exist as relatively short stand‐alone fragments that are synthesized, used for translation, and then later digested. The chirality of the sugars that compose a hairpin‐structured RNA minihelix (d‐ribose or l‐ribose) was shown to determine whether the amino acid loaded onto the RNA, the step before being incorporated into a new peptide, is right‐ or left‐handed.^[^ ^9^ ^]^ d‐ribose based RNA minihelices correspond to L‐form amino acids, both of which are dominant *in vivo*. R‐loops,^[^ ^10^ ^]^ in which a strand of RNA displaces one of the two strands of a DNA duplex and forms an RNA:DNA hybrid structure, are usually right‐handed. In larger RNA‐containing structures, such as the ribosome, RNA loops, and hairpins often fold together with complex topologies, whose chirality is not clearly defined.

Less common DNA secondary structures, that is, outside of A‐, B‐, or Z‐form, primarily exist only locally, for specific types of nucleotide sequences. G‐quadruplexes,^[^ ^11^, ^12^ ^]^ Holliday junctions,^[^ ^13^ ^]^ cruciforms,^[^ ^14^ ^]^ i‐motifs,^[^ ^11^, ^15^ ^]^ triple helices,^[^ ^16^ ^]^ and opening “bubbles”^[^ ^17^ ^]^ are all non‐standard DNA secondary‐structure patterns. A Holliday junction consists of two anti‐parallel B‐DNA helices that cross in a right‐handed way.^[^ ^18^ ^]^ Cruciforms and i‐motifs can typically only form for palindromic sequences. A DNA cruciform exists either in an X‐shaped conformation, similar to a Holliday junction, or in an extended hairpin.^[^ ^19^ ^]^ Local unpairing of double‐stranded DNA (dsDNA) occurs at transcription and replication initiation sites and may involve the formation of slipped‐strand DNA structure, which was experimentally observed for di‐ or tri‐nucleotide repeat‐sequence DNA.^[^ ^20^ ^]^

The extent to which structures other than a right‐handed DNA double‐helix form in vivo, and their significance, is still under debate.^[^ ^21^ ^]^ Once primarily considered as structural oddities, Z‐DNA, R‐loops, and intercalated DNA structures are now recognized to have some biological functions. Left‐handed Z‐DNA serves as a signal and binding‐partner for the enzyme ADAR1,^[^ ^22^ ^]^ which in turn edits double‐stranded RNA, thereby limiting immune responses. Z‐DNA's ability to regulate this cell‐signaling pathway offers an avenue to combat a number of medical conditions,^[^ ^23^ ^]^ including viral infections^[^ ^24^ ^]^ and cancer.^[^ ^25^ ^]^ If persistent, an R‐loop can disrupt DNA transcription and replication processes^[^ ^26^ ^]^ whereas synthetically stabilized R‐loops have been considered for the selective inactivation of tumor cells.^[^ ^27^ ^]^ In vivo experiments found intercalated DNA structures (i‐motifs), such as the one shown in Figure 1c, to form near promoter and telomeric regions of the human genome.^[^ ^28^ ^]^

The DNA of eukaryotes and archaea associates with histone or histone‐like proteins in arrangements displaying local chirality. In a eukaryotic nucleosome (Figure 1d), double‐stranded DNA (dsDNA) superhelically winds around a histone octamer protein core. The superhelical winding is left‐handed in the canonical case, but experiment^[^ ^39^, ^40^, ^41^ ^]^ and simulation^[^ ^42^ ^]^ have indicated that histone assemblies can switch between left‐ and right‐handed superhelical states, which could be regulated, in part, by chaperone proteins.^[^ ^43^ ^]^ A canonical nucleosome exhibits pseudo two‐fold symmetry about its dyad,^[^ ^44^ ^]^ structural symmetry that can be broken by spontaneous unwrapping and rewrapping of the surrounding DNA, characterized in experiment^[^ ^45^, ^46^ ^]^ and simulation.^[^ ^47^, ^48^, ^49^ ^]^ In a crystallographic study,^[^ ^34^ ^]^ the histones of archaea, evolutionary precursors to the same proteins in eukaryotes, were found to form a continuous left‐handed superhelical ramp with DNA (Figure 1e).

Beyond the scale of single nucleosomes, a poly‐nucleosomal array could also exhibit chirality. However, the specific arrangement of consecutive nucleosomes, and the extent to which chromatin has a well‐defined higher‐order structure, remains debatable. A left‐handed helical pattern, referred to as the 30‐nm fiber,^[^ ^50^ ^]^ has long been considered, with some experiments now suggesting it is not a prominent local form for chromatin.^[^ ^51^ ^]^ Nevertheless, another set of experiments^[^ ^52^ ^]^ indicates that helical fibers of stacked nucleosomes are common, with right‐handedness favored over left. A coarse‐grained simulation study^[^ ^53^ ^]^ indicates that nucleosome core particles stack on top of one another, slightly favoring a left‐handed helical arrangement.

Supercoiling—a single DNA duplex coiling upon itself or multiple duplexes braided together—can either be toroidal, as it is in a nucleosome or archaeal superhelix, or plectonemic, a loop twisted upon itself, schematically shown in Figure 1f. Negatively supercoiled DNA (underwound), which is more common than positive, can lead to a right‐handed plectonemic structure. Plectonemes have long been known to exist within circular bacterial plasmids, stabilized by histone‐like proteins that bend and bridge the DNA^[^ ^54^ ^]^ and regulated by topoisomerases and RNA polymerases.^[^ ^55^ ^]^ In vitro experiments and theoretical modeling indicate that the localization of plectonemes on a DNA molecule is controlled by the DNA sequence through sequence‐dependence of its local intrinsic curvature.^[^ ^56^ ^]^ The prevalence and significance of plectonemes in eukaryotes is uncertain. One hypothesis is that topologically associating domains, the mega‐base pair building blocks of eukaryotic chromosomes, are themselves supercoils.^[^ ^57^, ^58^ ^]^

Throughout much of the cell‐cycle, DNA is pulled or twisted as a result of the processes fundamental to life. During DNA transcription, for example, RNA polymerase (RNAP) was shown to follow a straight path along dsDNA's helical axis, not a spiraling one that would match the minor or major groove. Therefore, RNAP's movement during this process induces local, torsional stress: positive DNA supercoiling occurs downstream, and negative supercoiling upstream (Figure 1g). Left‐handed Z‐DNA can form transiently immediately upstream of RNAP. Negative DNA supercoiling stimulates eukaryotic nucleosome formation, whereas positive supercoiling strongly inhibits it.^[^ ^59^ ^]^ Kim and co‐workers found^[^ ^60^ ^]^ transcription‐induced DNA supercoiling to affect the activity of distant RNAPs bound to the same DNA, with supercoiling cancellation and accumulation enhancing and reducing, respectively, the RNAP activity.

The overall conformation of genetic material within viruses and single‐celled eukaryotes can exhibit chirality as well. Illustrated in Figure 1h, a cyro‐EM study^[^ ^36^ ^]^ demonstrates that the highly condensed dsDNA genome of herpes simplex virus 1 adopts a chiral spooled conformation, even though the composition of its protein capsid is symmetric. Retrovirus MS2^[^ ^61^ ^]^ displays chirality, in part, because of the asymmetric construction of its protein capsid. For the tobacco mosaic virus,^[^ ^62^ ^]^ another retrovirus, the coat protein self‐assembles around the RNA into a rod‐like helical structure, consisting of 16.3 proteins per helical turn, with its chirality depending on that of the RNA. On an even larger scale, the chromatin of unicellular dinoflagellates is arranged into a series of arches (Figure 1i) indicating chiral liquid crystalline structure.^[^ ^37^, ^38^ ^]^

## 3 Synthetic Chiral DNA Systems

### 3.1 Liquid Crystal Phases of Dense DNA Solutions

**Figure 2:**
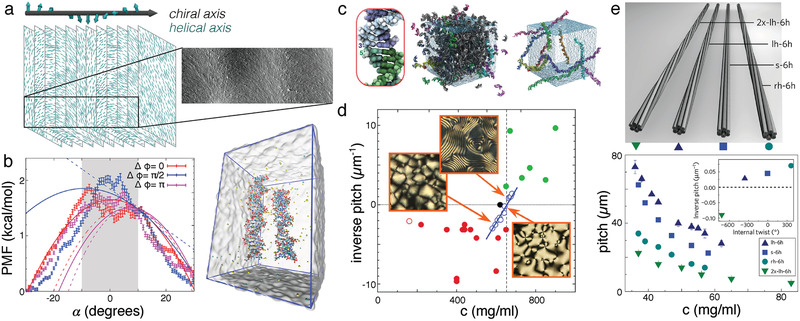
Chiral LC phases of dense DNA solutions. a) Schematic representation of the chiral nematic LC phase. The helical axes of the DNA molecules (cyan lines) are aligned to axes perpendicular to the chiral axis of the liquid crystal. As one translates along the chiral axis (gray arrow), the helical axis rotates (cyan axis). The rotation of the LC axis provides a distinct pattern in electron microscope images (right). Adapted with permission.^[^ ^66^ ^]^ Copyright 1996, Elsevier. b) All‐atom simulations reveal potential of mean force (PMF) between DNA double helices placed 26 Å apart as function of crossing angle *α*.^[^ ^67^ ^]^ The image depicts the simulation system containing a pair of 24‐bp DNA duplexes surrounded by 150 mM NaCl solution. Reproduced with permission under the terms of the Creative Commons Attribution 3.0 license.^[^ ^67^ ^]^ Copyright 2017, IOP Publishing. c) Filamentation of dsDNA through end‐to‐end stacking observed in all‐atom MD simulations.^[^ ^68^ ^]^ The left panel depicts an isolated pair of 10‐bp DNA helices after collapse into an end‐to‐end complex. The center panel shows a system containing 458 10‐bp DNA fragments with randomly generated initial positions. The right panel shows the long filaments that spontaneously formed during the 260‐ns simulation. Adapted with permission.^[^ ^68^ ^]^ Copyright 2012, Oxford University Press. d) The chirality of LC phases formed from short DNA fragments can be controlled by the sequence of the DNA. Each solid dot shows the critical concentration and helical wave vector for a DNA sequence (8–20 bp). The magenta and green dots indicate sequences giving left‐ and right‐handed LC chirality, respectively. The open magenta dot corresponds to long, 150 bp dsDNA. The open blue dots show the concentration dependence of the helical wave vector of a LC formed by the DNA of CGCGCCGGCGCG sequence. The black dot corresponds to an achiral DNA of AACGAATTCGTT sequence. Reproduced with permission.^[^ ^65^ ^]^ Copyright 2010, National Academy of Sciences. e) Six‐helix DNA bundles programmed using DNA origami to have variable internal twists (top) were condensed using dextran into LC phases with varying chiral properties (bottom).^[^ ^69^ ^]^ The pitch of the LC phase was seen to decrease with increasing concentration, as with LCs formed from short DNA duplexes (blue dots in (d)). Left‐ and right‐handed LCs were observed with the handedness of the LC correlating with the handedness of the bundle, but having a right‐handed bias, in contrast to LCs formed from bare DNA. Reproduced with permission.^[^ ^69^ ^]^ Copyright 2017, Springer Nature.

Double‐stranded DNA is a linear polymer that can be considered as stiff at a length scale of up to ≈50 nm, which is much greater than its width (≈2 nm). Hence, long fragments of DNA do not pack efficiently in an isotropic phase and tend to align along a common axis when condensed to sufficiently high density by crowding agents such as polyethylene glycol (PEG), forming a nematic liquid crystal (LC).^[^ ^63^, ^64^ ^]^ At sufficiently high densities of chiral molecules (including DNA), the broken symmetry of the underlying molecular interactions can manifest itself as a long‐range chiral order known as a chiral nematic liquid crystal phase, also referred to as a cholesteric phase. In such a phase, the molecules, on average, align along a common axis as with the nematic phase, but the helical axis rotates as one translates along an orthogonal chiral axis, as schematically shown in **Figure** **2**a. The chiral liquid crystal phases formed from DNA molecules have generally been found to be left‐handed.^[^ ^65^ ^]^

Many theoretical attempts have been made to relate the microscopic interactions between pairs of DNA molecules to the macroscopic properties of the liquid crystal phases.^[^ ^70^, ^71^ ^]^ The statistical mechanics linking the microscopic interactions to macroscopic behavior is inherently many‐bodied because of the presupposed close proximity of the DNA duplexes. Hence, the principle difficulty from a theoretical stand point is that one must integrate over, or adequately sample, the space of possible configurations of a dense many‐body system. This can, in principle, be done using analytical methods for extremely simple interaction models, but often computational approaches are used for more complex ones. To simplify the statistical mechanics of the system, a number of approximations are typically invoked, including treating each DNA helix as a rigid object. To our knowledge, all studies relating microscopic interactions to macroscopic phase behavior express the free energy of the system as an expansion of the many‐body interactions whose higher‐order terms are eventually truncated.

After applying the above approximations, the resulting expression for the free energy consists of configuration integrals that can be evaluated directly, usually after invoking additional approximations, or using density functional theory, to obtain the phase behavior of the system. A thorough description of the various theoretical and computational studies of DNA LC phase behavior was presented in Tortora and Doye.^[^ ^72^ ^]^ A significant result from these studies was the observation that steric interactions lead to a left‐handed twist, whereas electrostatics promote a right‐handed twist.^[^ ^70^ ^]^ However, all‐atom simulations found the free energy difference for arranging the DNA in left‐handed and right‐handed junctions to be rather small, (Figure 2b),^[^ ^67^ ^]^ suggesting that LC‐phase prediction from studies using rigid coarse‐grained models should be interpreted with caution. In addition, it has been argued that the very weak nature of chiral interactions renders the results of LC‐phase prediction studies sensitive to the numerical methods used to determine the free energy.^[^ ^72^ ^]^ A rigid‐duplex version of the oxDNA model^[^ ^73^, ^74^ ^]^ was used to evaluate the configurational integrals for 146 base pair (bp) DNA duplexes while monitoring and controlling the statistical error.^[^ ^72^ ^]^ These state‐of‐the‐art simulations indicated that the chiral LC phase formed by such rigid helices is very weakly right‐handed regardless of ion concentration—a result that is inconsistent with experiments that indicate weakly left‐handed chiral phases. Performing the DFT calculation using two distinct curved configurations of a DNA molecule resulted in very strongly chiral LC phases of opposite handedness. The sensitivity to conformation was shown to preclude reliable convergence of the DFT procedure when a flexible DNA model was considered, preventing the prediction of the helical pitch of the liquid crystal phase.

In 2007, the Clark and Bellini groups sent ripples through the DNA community by demonstrating that LC phases could be formed not only with relatively long DNA polymers (above ≈ 100 bp), but also from extremely short double‐helices (down to 6 bp).^[^ ^75^ ^]^ All‐atom simulations performed shortly thereafter confirmed the conclusion that the short, blunt‐ended DNA fragments could spontaneously stack via base‐stacking interactions to form long filaments needed to precipitate LC phases^[^ ^68^ ^]^ (Figure 2c). Specifically, the simulations found a preference for pairs of DNA helices to stack with a continuous 5′‐to‐3′ direction of the backbone across the junction so that aggregates resembled long DNA double helices with periodic nicks and occasional defects.

Subsequent experiments from the Clark and Bellini groups demonstrated that chiral nematic LC phases could be formed from dense mixtures of short DNA fragments.^[^ ^65^ ^]^ The groups further quantified the dependence of the phase behavior on DNA length and sequence. Although one might naïvely speculate that the geometry of end‐to‐end‐stacked DNA duplexes is insensitive to DNA sequence, experiments demonstrated that some sequences resulted in left‐handed chiral LC phases, whereas others resulted in right‐handed ones (Figure 2d). This observation emphasizes the remarkable sensitivity of chiral LC phase behavior to small differences in the microscopic interactions between molecules (or linear aggregates of molecules as for end‐to‐end stacked DNA fragments), a property that makes computational prediction of LC phases challenging. Nevertheless, the De Michele, Bellini, and Ferrarini groups were able to apply DFT theory using a coarse‐grained model of short DNA fragments to predict the cholesteric phase behavior and the concentration dependence of the pitch from the structural features of the molecules forming the condensate.^[^ ^76^ ^]^ Clark and Bellini have also experimentally studied the LC phases formed by mixtures of ordinary D‐DNA 12‐mers and mirror image L‐DNA 12‐mers of the same nucleotide sequence.^[^ ^77^ ^]^ L‐DNA^[^ ^78^ ^]^ differs from ordinary D‐DNA only in the stereoscopic arrangement of a few atoms in the sugar ring of DNA, yet L‐DNA is conspicuously absent in biology. The dependence of the pitch on the fraction of D‐DNA in the mixture was found to be symmetric about 50%. However, the chirality of the LC phase depended on the terminal chemistry, with sticky‐ended duplexes forming a left‐handed phase and blunt‐ended duplexes forming a right‐handed phase when the fraction of D‐DNA was greater than 50%. This result again emphasizes the sensitivity of chiral DNA LC phases to the details of the molecular interactions in the system.

The Dietz group studied chiral liquid crystals formed by six‐helix bundles of DNA^[^ ^69^ ^]^ built using a technique known as DNA origami.^[^ ^79^ ^]^ The bundles of DNA were programmed to have different amounts of twist, varying from left‐handed to neutral and to right‐handed. They found that the pitch along the chiral axis depended non‐linearly on the amount of twist in the underlying DNA structure (Figure 2e). Although increasing the right‐handed twist of underlying structures increased the right‐handedness of the resulting chiral structures, straight and even weakly left‐handed DNA nanostructures were seen to produce right‐handed chiral structures.

The Doye group analyzed the cholesteric LC phases of the above six‐helix bundle systems using the oxDNA model to offer a microscopic description of the chiral arrangement of the bundles,^[^ ^80^ ^]^ and providing a platform to systematically study the relationship between molecular interactions and cholesteric behavior. First, it was noted that the straight, ground state configuration of the bundles produced phases of opposite chirality compared to those observed in experiment. Then, when fluctuations of the bundles were considered, individual bundles were seen to adopt, on average, superhelical configurations that opposed the designed twist of the bundle (e.g., a bundle with left‐handed twist would adopt an overall right‐handed helical geometry). The free energy of two interacting bundles was then dominated by the superhelical writhe rather than the programmed twist, resulting in cholesteric pitches similar to those observed in experiment. From a methodological standpoint, it is interesting to note that the ratio of the contour length to the persistence length was much larger for the bundles than for the 146‐bp duplexes previously studied by the Doye group,^[^ ^72^ ^]^ a property that permitted convergence of the DFT algorithm when fluctuations were considered.

Finally, it is interesting to note that liquid crystals of DNA can be mineralized by the addition of positively charged molecules that contain silicon.^[^ ^81^, ^82^ ^]^ Such crystalline structures can be studied using electron microscopy and presumably have distinct mechanical properties compared to DNA, being substantially more rigid. The addition of cations can be used to induce chiral assembly into nanoscale impeller‐like structures with tunable handedness, depending on the pH and temperature of the solution.^[^ ^82^ ^]^ These systems again emphasize the sensitivity of chiral self‐assembly on the microscopic details of inter‐DNA interactions.

### 3.2 Programmable Chirality in Self‐Assembled DNA Nanostructures

**Figure 3:**
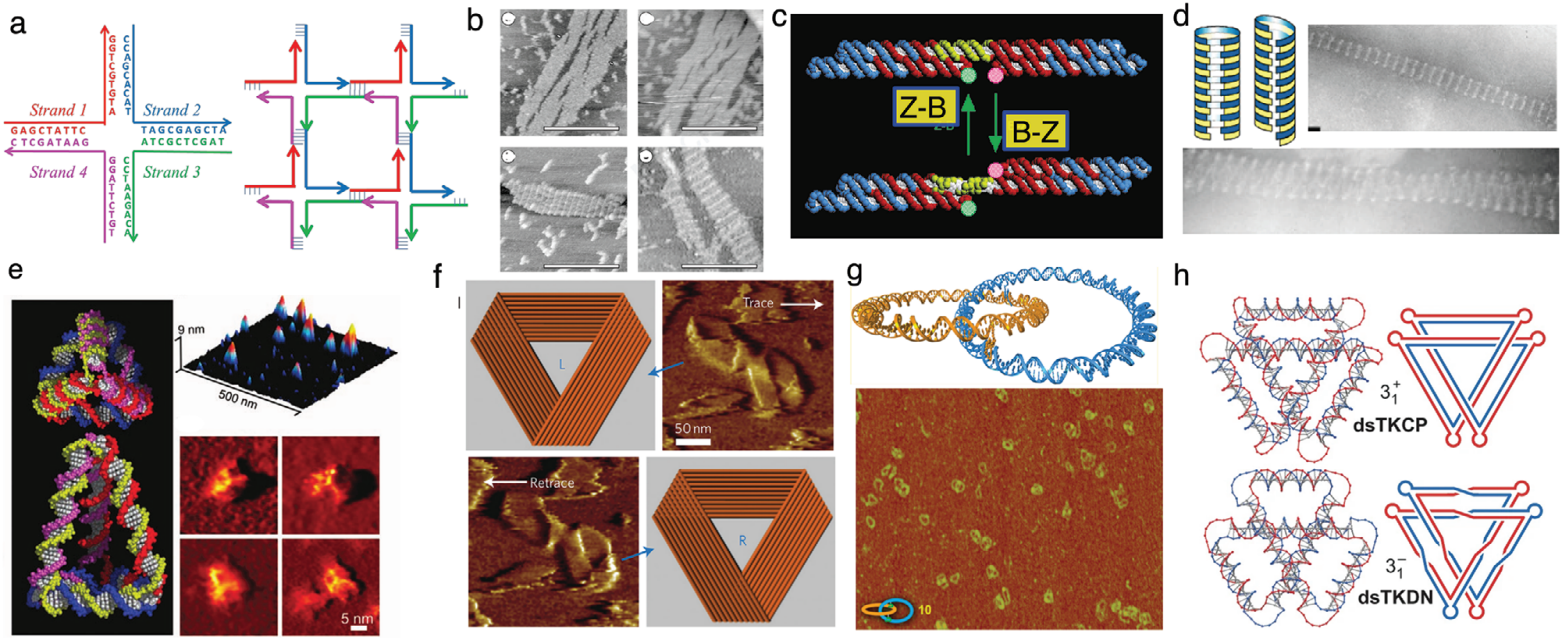
Topological DNA nanostructures. a) Schematic representation of a Holliday junction (left) and self‐assembly of many such junctions into a periodic lattice using the sticky‐ended cohesion principle (right). b) AFM images of double crossover (DX) DNA lattices; scale bars: 300 nm. Reproduced with permission.^[^ ^86^ ^]^ Copyright 1998, Springer Nature. c) A nanomechanical switch that changes its conformation as a dsDNA duplex linker (yellow) undergoes a B‐to‐Z (or vice versa) transition in response to modulation of the solution conditions. Reproduced with permission.^[^ ^88^ ^]^ Copyright 1999, Springer Nature. d) Schematics and TEM images of achiral and chiral DNA nanotubes obtained by wrapping DX tiles along different basis vectors. Reproduced with permission.^[^ ^89^ ^]^ Copyright 2004, American Chemical Society. e) Design and AFM images of diastereomeric DNA tetrahedron. Reproduced with permission.^[^ ^90^ ^]^ Copyright 2005, American Association for the Advancement of Science. f) Schematics and AFM images of left‐handed (top) and right‐handed Möbius strips made from DNA. Reproduced with permission.^[^ ^91^ ^]^ Copyright 2010, American Chemical Society. g) Design and AFM images of topologically catenated DNA circles. Reproduced with permission.^[^ ^92^ ^]^ Copyright 2011, Springer Nature. h) Schematics of dsDNA knots made from four‐way DNA junctions. Reproduced with permission.^[^ ^93^ ^]^ Copyright 2016, Springer Nature.

Prior to the inception of the DNA origami method, DNA nanostructures were most commonly constructed using self‐assembled 2D arrays of DNA molecules, so‐called DNA tiles.^[^ ^83^, ^84^ ^]^ A DNA tile typically features a double crossover (DX) junction, which is a fixed‐geometry variant of a naturally occurring Holliday junction^[^ ^85^ ^]^ (**Figure** **3**a). Large periodic arrays of such junctions can be assembled using the sticky‐ended cohesion principle^[^ ^86^, ^87^ ^]^  (Figure 3b), where short overhands of ssDNA form a DNA duplex upon hybridization with a complimentary ssDNA overhang.

One of the most exciting early developments in the field of DNA self‐assembly was the demonstration of a DNA nanostructure that could undergo a reversible change of its global conformation, driven by a B‐to‐Z transition in a DNA duplex connecting two DX tiles^[^ ^88^ ^]^ (Figure 3c). Using an approach analogous to folding a 2D carbon sheet of graphene into a carbon nanotube, DNA nanotubes up to several micrometers in length were constructed by wrapping DX tile arrays.^[^ ^94^, ^95^, ^96^, ^97^ ^]^ Turberfield and co‐workers synthesized DNA nanotubes of different chiralities by wrapping DX tiles along different basis vectors of the DX lattice^[^ ^89^ ^]^ (Figure 3d). The resulting DNA nanotubes had both the intrinsic chirality of the DNA molecules and an additional mesoscopic chirality determined by the chosen basis vector. Yan and co‐workers used L‐DNA to build left‐handed DNA arrays and to control the chirality of the DNA nanotubes produced by folding of the arrays.^[^ ^98^ ^]^

Another class of self‐assembled DNA objects are wireframe and tensegrity structures,^[^ ^99^ ^]^ such as cubes,^[^ ^100^ ^]^ tetrahedra,^[^ ^90^ ^]^ octahedra,^[^ ^101^, ^102^ ^]^ icosahedra,^[^ ^103^, ^104^ ^]^ knots,^[^ ^92^, ^105^, ^106^ ^]^ and rotaxanes.^[^ ^107^ ^]^ Some polyhedral DNA nanostructures can fold into distinct stereoisomeric forms,^[^ ^102^ ^]^ which is undesirable for their potential applications that require precise arrangement of functionalized groups, such as plasmonics.^[^ ^108^ ^]^ Goodman et al. demonstrated a method for designing a DNA tetrahedron that promotes self‐assembly of one diastereomer over another^[^ ^90^ ^]^ (Figure 3e). Using the asymmetric three‐point star DNA motifs, Mao and co‐workers synthesized triangular DNA prisms whose chirality could be set by controlling the angles at the DNA junctions.^[^ ^109^ ^]^

DNA can also be used to construct topologically closed and interlocked assemblies, many of which are inherently chiral. The Yan lab engineered various types of DNA origami nanostructures with non‐trivial topologies,^[^ ^110^ ^]^ including both, left‐ and right‐handed Möbius strips^[^ ^91^ ^]^ (Figure 3f). Schmidt and Heckel developed a process for building interlocked DNA minicircles^[^ ^92^ ^]^ (Figure 3g), which can form the basis for synthetic nanomotors. Liu et al. synthesized trefoil knotted structures by taking advantage of the inherent chiral topology of the four‐way dsDNA junction^[^ ^93^ ^]^ (Figure 3h). Yan and co‐workers constructed knotted nucleic acid nanostructures using single‐stranded DNA and RNA, achieving crossing numbers of up to 57.^[^ ^106^ ^]^

**Figure 4:**
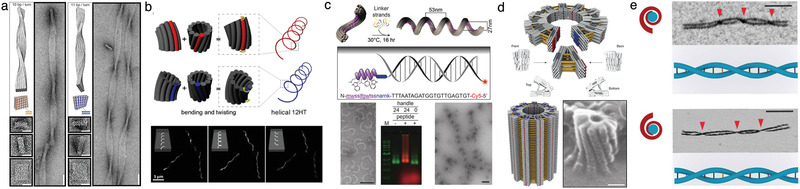
Self‐assembled DNA nanostructures of programmable twist. a) TEM images of twisted DNA origami bundles. The global twist was induced by changing the number of base pairs per turn of a DNA helix. Reproduced with permission.^[^ ^111^ ^]^ Copyright 2009, American Association for the Advancement of Science. b) Schematics of the induced chirality (top) and its characterization using fluorescence micrography (bottom) in a tile‐based DNA helix tube upon the addition or deletion of a base pair. Reproduced with permission.^[^ ^112^ ^]^ Copyright 2017, American Chemical Society. c) Schematics of the bottom‐up self‐assembly of a left‐handed DNA nanospring from 24‐helix DNA bundles labeled with membrane binding molecules (top) and TEM images of the assembled structure (bottom); scale bar: 50 nm. Reproduced with permission.^[^ ^113^ ^]^ Copyright 2018, John Wiley & Sons. d) Schematics and helium‐ion micrographs of a gigadalton, 450 nm diameter tube assembled from V‐bricks; scale bar: 50 nm. Reproduced with permission.^[^ ^114^ ^]^ Copyright 2017, Springer Nature. e) Schematics and AFM images of the right‐handed and left‐handed meta‐DNA nanostructure; scale bar: 100 nm. Adapted with permission.^[^ ^115^ ^]^ Copyright 2020, Springer Nature.

Presently, the DNA origami method^[^ ^79^ ^]^ is the most popular approach for creating self‐assembled DNA nanostructures. In the DNA origami method, short synthetic “staple” DNA strands are used to guide folding of a long “scaffold” DNA strand into a custom 3D shape.^[^ ^79^, ^116^ ^]^ The method's success is due in part to the computer aided design tool cadnano,^[^ ^117^ ^]^ which simplifies the design process by providing a lattice‐based design framework and a graphical user interface. The equilibrium shape of a complex 3D DNA origami nanostructure was found to be sensitive to the distance between the crossovers connecting adjacent helical domains.^[^ ^111^ ^]^ The Shih lab varied the number of base pairs between the crossovers to build right‐handed and left‐handed DNA bundles (**Figure** **4**a).^[^ ^111^ ^]^ The Liedl lab demonstrated that the chirality of a DNA nanotube can be regulated by adding base pairs on one face of the tube and removing them from the other^[^ ^112^ ^]^ (Figure 4b).

In biological systems, lipid bilayer‐enclosed compartments are used to seclude and regulate biochemical processes. There has been a lot of interest in using DNA to create membrane‐embedded structures^[^ ^118^, ^119^ ^]^ that behave as channels^[^ ^120^, ^121^ ^]^ and transporters.^[^ ^122^, ^123^ ^]^ Larger, membrane enclosed structures have been assembled by functionalizing a DNA scaffold with hydrophobic lipid anchors.^[^ ^124^ ^]^ Lin and co‐workers created chiral DNA origami “springs”^[^ ^113^ ^]^ that, upon polymerization, produced tubular offshoots from a lipid vesicle (Figure 4c). Such structures could be used to create passageways to direct the chemical traffic between lipid vesicles.^[^ ^125^ ^]^

In 2012, the Dietz group utilized a globally chiral DNA origami design to allow the TEM images of the object to be easily aligned, providing the first high‐resolution cryo‐EM reconstruction of a 3D DNA origami object.^[^ ^126^ ^]^ The reconstruction revealed an unintended, slight global twist in the structure, which was confirmed by simulation^[^ ^127^ ^]^ and has since been observed as a general feature of standard DNA origami designs. Often this chirality is regarded as a nuisance. For example, the Dietz group found that the unintended twist limited hierarchical self‐assembly, requiring the redesign of triangular V‐brick components to remove the residual twist and allow self‐assembly of gigadalton‐sized DNA tubes^[^ ^114^ ^]^ (Figure 4d). Upon imaging, the gigadalton origami structures revealed an unintended twist of unknown origin.

In order to scale up the size of DNA nanostructures, the Yan group developed a meta‐DNA strategy which uses six‐helix DNA origami bundle “backbone” functionalized with sticky strand “bases” as a magnified analogue of a single‐stranded DNA molecule.^[^ ^115^ ^]^ They synthesized left‐ and right‐handed meta‐DNA by programming the chiral arrangement of the constituent meta‐DNA bases (Figure 4e). The right‐handed double‐stranded meta‐DNA was found to be almost five times more rigid as compared to its left‐handed analogue, which suggests that the chirality plays an important role in the mechanical properties of such structures.

## 4 Applications

### 4.1 DNA‐Based Plasmonic Nanostructures

Chiral molecules typically exhibit different absorption of left‐ and right‐handed circularly polarized light—a property known as circular dichoism (CD), which has been utilized extensively to characterize chiral molecular structures.^[^ ^133^ ^]^ When the difference between absorptions of left‐ and right‐handed polarized light is plotted as a function of wavelength, a pattern of peaks and valleys emerges due to the chirality of the underlying molecules that acts as a sort of fingerprint for the molecule. Most media that exhibit a CD signal have a very weak optical response.^[^ ^134^ ^]^ However, a weak optical response can be amplified using plasmonic nanostructures.^[^ ^135^, ^136^ ^]^ For example, Kneer et al. showed an enhanced CD response of a DNA origami sheet when it was placed between two gold nanoparticles.^[^ ^137^ ^]^

A plasmon is a quantum of collective free electron gas oscillation in metal,^[^ ^138^ ^]^ which can be excited by light of a specific geometry‐dependent (resonant) frequency. If the wavelength of the excitation is similar to or longer than the size of the metal particle (the particle size is typically tens to hundreds of nanometers), the plasmon is localized to the particle's surface. When a plasmon is excited, the electron gas in the particle behaves like a simple dipole oscillating parallel to the direction of the oscillating electric field, enhancing the electric field within one wavelength of the particle's surface (the near‐field zone) and increasing the particle's cross sections for both scattering and absorption. If another particle with similar resonance frequency is present in the near‐field zone, a collective hybrid mode can be formed. Chiral plasmonic modes can form in either handed nanoparticle groups or in chiral‐assembled groups of individual resonant nanoparticles.^[^ ^139^ ^]^ This kind of optical response is much stronger than those of chiral molecules and is detectable in the visible light regime, which is highly desirable for sensing applications.

Fabrication of chiral plasmonic devices using traditional lithography methods can be expensive and error prone, in particular if the devices have complex 3D geometries. Being robust, precise, and programmable, self‐assembly of DNA has allowed the fabrication of complex plasmonic nanostructures in solution.^[^ ^108^, ^129^, ^132^ ^]^ DNA molecules can either link metal nanoparticles into a prescribed geometrical shape^[^ ^108^, ^128^ ^]^ or serve as a template for metal nanoparticle assembly.^[^ ^129^, ^131^, ^140^ ^]^ Self‐assembled DNA nanostructures have also been used to build entirely metallic nanostructures with designed plasmonic properties.^[^ ^141^ ^]^ Note that, in the plasmonic structures discussed here, the DNA serves as a template upon which plasmonic particles are arranged in a chiral manner.

**Figure 5:**
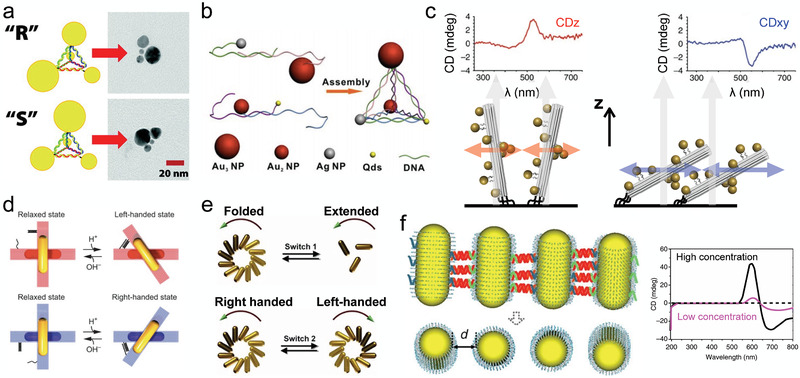
DNA‐based chiral plasmonic nanostructures. a) DNA pyramid decorated with four different‐sized gold nanoparticles. Reproduced with permission.^[^ ^108^ ^]^ Copyright 2009, American Chemical Society. b) DNA pyramid containing four different types of particles at its vertices. Reproduced with permission.^[^ ^128^ ^]^ Copyright 2012, American Chemical Society. c) Switching CD response of a gold–DNA nanostructure by dehydration. When solvated, DNA rods decorated with gold nanoparticles preferentially align normal to the surface (left). The direction of the CD response switches by 90° upon drying (right). The propagation direction of a circularly polarized light is indicated by arrows. Reproduced with permission.^[^ ^129^ ^]^ Copyright 2013, Springer Nature. d) Chiral plasmonic systems containing a pH‐sensitive DNA lock. The pH value controls the population of the right‐handed and left‐handed structures. Reproduced with permission.^[^ ^130^ ^]^ Copyright 2017, American Association for the Advancement of Science. e) Chiral plasmonic nanostructure can be switched between folded and extended states (top) and between right‐ and left‐handed states (bottom). The arrangement of gold nanoparticles attached to a DNA origami structure is reconfigured by the addition of DNA strands. Reproduced with permission.^[^ ^131^ ^]^ Copyright 2018, American Chemical Society. f) Plasmonic sensor of DNA concentration. Linking gold nanorods by analyte DNA tilts the nanorods with respect to each other (left), which is detected by measuring CD spectra (right). Reproducedand adapted under the terms of the Creative Commons CC‐BY license.^[^ ^132^ ^]^ Copyright 2013, Springer Nature.

One of the earliest examples of a plasmonic DNA nanostructure was a DNA pyramid that contained four different‐sized gold nanoparticles placed at the pyramid's vertices by means of covalently linked DNA strands (**Figure** **5**a).^[^ ^108^ ^]^ Govorov and co‐workers theoretically showed that gold nanoparticles arranged on such pyramid‐shaped objects, as well as on helices, should exhibit a strong CD response in the visible light regime, exceeding the response of natural chiral molecules. Yan et al. experimentally measured the CD response from a pyramid DNA nanostructure,^[^ ^128^ ^]^ showing that intensity of the CD spectra can be precisely controlled through spatial arrangement of different types of nanoparticles at the pyramid's vertices (Figure 5b). A pyramid‐like arrangement of four nanoparticles was also realized using a sheet‐like DNA origami bundle by attaching three gold nanoparticles to one side of the DNA origami sheet and the fourth to the other side, creating a chiral arrangement.^[^ ^140^ ^]^ Helical metal–DNA metamolecules were assembled by Kuzyk and co‐workers by decorating a 24‐helix DNA origami rod with 10 gold nanoparticles arranged in a helical pattern.^[^ ^142^ ^]^ The handedness of the nanoparticle arrangement in such structures was found to flip the sign of the CD angle shift in the visible light regime.

Incorporating optically active elements into conformationally dynamic DNA nanostructures makes it possible to modulate the optical activity of such nanostructures by external stimuli. A conformational transition in a DNA nanostructure can be triggered by either changing the nanostructure's environment or by using dynamic DNA nanotechnology design motifs, including lock‐and‐key DNA strands and toehold‐mediated strand displacement.^[^ ^131^ ^]^ Schreiber et al.^[^ ^129^ ^]^ used helical metal–DNA nanostructures to construct systems that modulated optical response upon drying or rehydration (Figure 5c). In those systems, drying or rehydration altered the conformation of the nanostructures with respect to the surface they were attached to, switching the direction of the CD response from normal to the surface under wet conditions to parallel to the surface upon drying. In a seminal study, Kuzyk et al. assembled two 14‐helix DNA origami bundles, each decorated with a gold nanorod, in the shape of a cross and used auxiliary DNA strands to switch the orientation of the bundles from parallel to normal and vice versa.^[^ ^143^ ^]^ A similar principle was employed to realize pH‐switchable nanostructures,^[^ ^130^, ^144^ ^]^ where pH‐modulated affinity of the TAT/CGC triplets in the DNA locking strands (Figure 5d). Coating gold rods with a layer of silver was shown to greatly increase the optical response of the nanorod systems.^[^ ^145^ ^]^ Lan et al.^[^ ^131^ ^]^ demonstrated switching between more than two chiral states using a structure in which multiple gold nanorods were attached to a V‐shaped DNA origami template (Figure 5d). A folded structure could be converted to an extended structure by adding lock‐and‐key DNA strands (switch 1 in Figure 5e), and also from left‐handed to right‐handed conformations via a toehold‐mediated strand displacement reaction (switch 2 in Figure 5e). In another approach,^[^ ^146^ ^]^ the mutual orientation of several metal nanorods attached to multiple layers of DNA origami rods and sheets was controlled by means of blocker and activator strands, realizing eight distinct plasmonic stereoisomers.

Conjugating metal nanoparticles to a DNA scaffold has made it possible to amplify changes in the CD response caused by molecular interactions for biosensing applications. One such sensor was described by Ma et al.,^[^ ^132^ ^]^ where self‐assembly of gold nanorods was mediated by hybridization of the analyte strand to strands conjugated with the nanorods (Figure 5f). In the assembly, connecting one nanorod to the other via DNA duplexes was found to rotate one nanorod with respect to the other, giving the system a CD response. The amplitude of the CD response was found to increase with the concentration of the DNA analyte and allow detection of the DNA analyte present at attomolar concentrations.

### 4.2 DNA Spintronics

**Figure 6:**
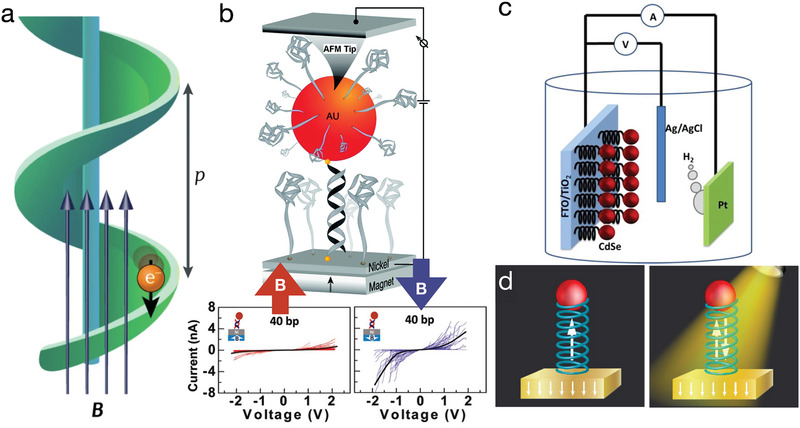
Chiral‐induced spin selectivity. a) Mechanism of chiral spin selection with a helical molecule. Reproduced with permission.^[^ ^147^ ^]^ Copyright 2019, Springer Nature. b) Chiral spin selection using dsDNA. Top schematic: A permanent magnet is placed under a Ni substrate, forcing the spin of the substrate to align. An AFM tip placed in contact with a gold nanoparticle is used to measure the current between the nanoparticle‐DNA‐substrate.^[^ ^148^, ^149^ ^]^ Bottom two panels: experimental measurements on a system shown in the schematic. Because DNA's chirality only allows one type of spin to transport from substrate to nanoparticle, and only the other type of spin from nanoparticle to substrate, the current passing from the substrate through dsDNA to the AFM tip is larger when the magnetic field is pointing down. Adapted with permission.^[^ ^148^, ^149^ ^]^ Copyrights 2011 and 2012, American Chemical Society. c) Chiral molecule‐assisted water splitting. The presence of chiral molecules on the anode increases the efficiency of producing H_2_. Reproduced with permission.^[^ ^150^ ^]^ Copyright 2015, American Chemical Society. d) Hypothetical memory device built using chiral molecules. Because of the spin selectivity of chiral molecules, the substrate is now magnetized by injecting one spin and pulling out the opposite spin. Chiral DNA nanostructures could potentially be used to build this type of a memory device. Reproduced with permission.^[^ ^147^ ^]^ Copyright 2019, Springer Nature.

The intrinsic curvature of a chiral molecule ensures that, as an electron moves through it, an effective magnetic field is generated. This magnetic field acts on the magnetic moment of the electron, and as a result, the tunneling probability through the chiral molecule differs for electrons with opposite spin. This phenomenon is referred to as chiral‐induced spin selectivity (CISS),^[^ ^148^, ^151^, ^152^ ^]^ schematically shown in **Figure** **6**a. The physical origins of CISS can be traced back to the spin‐orbit coupling, which is a relativistic effect that arises from the magnetic torque exerted on the electron when it is orbiting around the nucleus.^[^ ^153^, ^154^ ^]^ When considering a 2D material with a broken symmetry, such as an interface, the currents of electron and spins become coupled through the Rashba effect,^[^ ^153^, ^154^ ^]^ which allows for manipulation of spin degrees of freedom via electric field.

Experiments have shown that dsDNA molecules can produce a measurable CISS effect^[^ ^148^, ^149^ ^]^ (Figure 6b). In these experiments, thiolated ssDNA molecules were absorbed onto a Ni substrate while complementary ssDNA strands were bound to gold nanoparticles. Upon hybridization of the stands, Ni–dsDNA–Au junctions were formed and their electrical conductivity was probed using AFM with a conductive tip operating in a contact mode.^[^ ^148^, ^149^ ^]^ A permanent magnet was used to magnetize the Ni substrate, creating two conduction bands in the Ni substrate: one band corresponding to the electrons with spin aligned with the magnetic field and the other aligned opposite to the magnetic field. The resulting *I*–*V* curves are shown in Figure 6b, bottom. When the spin orientation of the Ni substrate aligns with the favored transmission direction through the chiral molecule, the current is higher compared to when the Ni substrate is magnetized in the other direction. Note that the *I–V* curves are symmetric, which means the opposite‐spin electrons flow from gold to Ni when an opposite bias is applied. We note further that, in DNA spintronics, tunneling occurs between the aromatic orbitals of nucleobases; however, chiral molecules without aromatic groups also exhibit this effect. This kind of spin selectivity can happen anywhere a chiral potential exists.

A potential application of DNA's spin selectivity effect is electrochemical water‐splitting (Figure 6c). The separation of water, which is spin singlet (containing no unpaired electrons), into H_2_ (singlets) and O_2_ (naturally in a spin triplet state, with a total spin of one) is spin forbidden, and thus, this process is slow.^[^ ^147^ ^]^ Spin selection can help accelerate this process. In the experiments performed by Mtangi et al.,^[^ ^150^ ^]^ CdSe nanoparticles (red) were connected to TiO_2_ nanoparticles, and then the TiO_2_ nanoparticles were attached to the fluorine‐doped tin oxide (FTO) conducting electrode, serving as the anode. The Pt electrode served as the cathode, and hydrogen was produced from that side. Because of the presence of chiral molecules, electrons of only one spin state could be transferred from CdSe to titania. Thus, the spin orientation of holes in the CdSe nanoparticles was well defined. Since the oxygen atoms had the same unpaired spin, the probability of forming O_2_ was much higher than in a system containing no chiral molecules.^[^ ^150^ ^]^

Because of their switchability and long coherence time, spin systems are promising candidates for storing quantum information. Utilizing CISS to build a spin memory device is also possible, and has been experimentally realized for several (but not nucleic acid) chiral molecules.^[^ ^155^, ^156^, ^157^ ^]^  Figure 6d illustrates the design of a CISS‐based memory, which could potentially use dsDNA as a CISS filter molecule. The left panel of Figure 6d depicts an electrical memory, written and read out by the electric current, as conceived by Koplovitz et al.^[^ ^157^ ^]^ The ferromagnetic nanoparticle (red) is first magnetized in the direction anti‐aligned with the spin direction favoring transfer from substrate to nanoparticle. To write in the memory, a high cross‐molecule voltage is applied. Only one spin is allowed to pass through the chiral molecule to the substrate, and, thus, the nanoparticle's spin is flipped. To read out the information, a lower voltage is applied. Because the written bits' spin states are already occupied, the unwritten bits will have a lower resistance than the written bits. The right panel of Figure 6d shows a design of a single bit of optical memory, driven by circularly polarized light. When the nanoparticle is excited by light, the charge oscillates with the electric field, and creates a net spin‐transfer torque between the excited quantum dot and the ferromagnetic substrate, leaving a local magnetization in the substrate.^[^ ^147^, ^155^ ^]^ Ben et al. were able to read out this type of memory by measuring the Hall voltage across the substrate.^[^ ^155^ ^]^ While these two types of spin‐selective memories are silicon‐compatible, erasing information requires chemical replacement of the molecules with their enantiomers, which can be a highly inefficient process. Since methods for switching a DNA origami's chirality have already been developed, DNA origami constructs could potentially play a role in the future development of this type of memory device.

### 4.3 Other Emergent Application Areas

**Figure 7:**
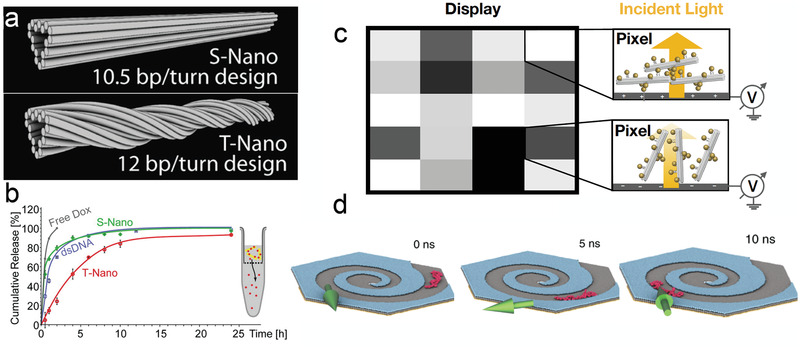
Emerging application areas. a) Straight (upper) and twisted (lower) DNA origami nanotubes.^[^ ^158^ ^]^ b) The release rate of drug Dox from straight (S) and twisted (T) nano‐designs. a,b) Adapted with permission.^[^ ^158^ ^]^ Copyright 2012, American Chemical Society. c) Hypothetical mechanism of an actively‐switchable display. Each pixel contains a collection of chiral plasmonic origami nanostructures tethered to a transparent electrode surface illuminated by circularly polarized light. A small voltage is applied to make the electrode surface negative or positive, causing the helix to orient parallel or orthogonal to the incident light, respectively. When the helix is parallel to the light, the absorption is greater, making the pixel appear dark. DNA origami nanostructure designs adapted with permission.^[^ ^129^ ^]^ Copyright 2013, Springer Nature. d) Delivery of DNA to a nanopore using a chiral step‐defect graphene guide. Adapted with permission.^[^ ^159^ ^]^ Copyright 2019, Springer Nature.

The control and customizability of DNA nanostructures makes them an attractive prospect for targeted drug‐delivery. Specifically, DNA origami‐based assemblies have served as vehicles for the delivery of anticancer drug doxorubicin (Dox), which intercalates between DNA bases, to infected cells.^[^ ^158^, ^160^, ^161^ ^]^ In one such study^[^ ^158^ ^]^ two rod‐like DNA‐origami nanostructures with different global designs (**Figure** **7**a) were used to deliver Dox to human breast cancer cells. The globally twisted structure was found to release the drug more slowly, compared to the straight origami‐based structure or freely diffusing Dox (Figure 7b). Thus, the kinetics of the drug release appears to be controlled by the global twist of the nanostructure, which open interesting possibilities for regulating drug release dynamically or conditioning the release speed on the presence of specific biomarkers.

Information could be stored or displayed with nanoscale precision using DNA origami as a building material. DNA origami's spin‐selectivity could be harnessed to build an array of bits, which could potentially serve as a memory device. Previous studies^[^ ^155^, ^157^ ^]^ used an array of short *α*‐helical peptides to fabricate such a device (Figure 6d). Another potential application is an actively‐switchable display (Figure 7c), which could be constructed using nanoparticles arranged using DNA origami into a chiral pattern to provide a strong CD response. When right‐handed light shines along the helical axis of a left‐handed helix, much of the light will be absorbed.^[^ ^129^ ^]^ If the same helix is rotated so that its helical axis is orthogonal to the light, the absorption will decrease. It was recently demonstrated that a DNA origami bundle can be reversibly switched between upright and flat configurations using an electrode.^[^ ^162^ ^]^ This mechanism could be used to electrically switch absorption states to make a display.

Last but not least, chiral nanostructures can be instrumental for delivery of individual biomolecules, such as DNA strands or unfolded proteins. Figure 7d illustrates one such possibility, where a spiral step defect in a multi‐layer graphene membrane guides the delivery of a single DNA strand to the center of the spiral.^[^ ^159^ ^]^ Molecular dynamics simulations and AFM experiments have established that the displacement of an adsorbed molecule over a step defect has a directional anisotropy and that such a molecule is much more likely to move along the step‐defect edge than across it. Thus, an external force that switches direction in a pattern that matches the chirality of the edge defect structure will bring the molecule to the center of the structure regardless of where the molecule is adsorbed, whereas applying the external force in the opposite chirality pattern will move the adsorbed molecule away from the center of the structure.

## 5 Conclusions and Outlook

From biology to nanotechnology, chirality is an intrinsic property of any DNA system. In biological systems, this chirality plays a major role in prescribing the preferred structure and dynamics to biological assemblies containing nucleic acids, the proteins they interact with, and the organization of genetic material within the cell. The most pertinent unanswered questions in this area relate to the very origins of life. Is it really a work of chance that life on Earth exclusively utilizes right‐handed nucleic acids or was that itself a product of natural selection? Future quests for traces of biological life on Mars and beyond may provide important clues to that. Another unchartered area is the structural organization of genomes in eukaryotic organisms, where we are only now starting to supplement biochemical and bioinformatic data with structural physical models. Finally, the practical utility of chiral structures in the macroscopic world is often to couple the motion along a linear path to a rotation about the path's axis. Interestingly, such coupling is rarely observed in biological systems, where molecular motors are found to take a linear path on a seemingly chiral track, such as in the motion of RNAP along a DNA duplex or the hand‐over‐hand motion of a kinesin on a microtubule.

The chiral LC phases formed by dense DNA mixtures provide a stringent test of our understanding of ordering in soft matter systems. Despite significant effort, the path by which the broken mirror symmetry of a molecule propagates from atoms to macroscopic material has only been partially charted. Strongly chiral, well‐studied, and programmable DNA systems provide the perfect testbed for studying the fundamental statistical mechanics of chiral LC phases. The ongoing theoretical, methodological, and experimental advancements in this area can be expected to result in a continuing stream of insights and, likely, new technologies that exploit the chiral phase behavior of DNA.

With regard to using DNA as a building material, the chirality of DNA is a mixed blessing. Because the building material is intrinsically chiral, the nanostructures based on DNA are constructed from chiral junctions arranged in chiral patterns, even when the goal is to develop a symmetric structure. The chirality of DNA and of the junctions have been exploited in many distinct ways to achieve diverse results, ranging from actuation to selection of a knotted stereoisomer. In larger DNA assemblies, sometimes the chirality of DNA manifests as an unwanted twist through a nanostructure. Other times, a custom chirality is intentionally introduced into the assembly. Whether desired or not, chirality is a fundamental attribute of DNA nanostructures that needs to be taken into account to achieve optimal functionality of a self‐assembled DNA system.

Harnessing chirality of DNA nanostructures for practical applications has been so far marked with some technological achievements, with the most promising applications being in the area of configurable plasmonic nanostructures and nanosensors. Although the chirality of DNA provides a natural mechanism for interacting with circularly polarized light, the majority of such applications use DNA either as a building material or as a sensitive transducer of molecular binding events into structural motion. In the field of spintronics, both the chirality of the DNA structure and the ability of DNA to self‐assembly into orderly larger‐scale structures could have a competitive advantage over solid‐state systems, which are difficult to assemble with the same precision and density at the nanoscale. If the past 20 years of DNA nanotechnology proves anything, it is that anything is possible.

## Conflict of Interest

The authors declare no conflict of interest.
